# Sovietization of sports in Czechoslovakia and the establishment of the institute of physical education and sport (1948–1953)

**DOI:** 10.3389/fspor.2026.1699191

**Published:** 2026-04-22

**Authors:** Marek Waic, Václav Pechman

**Affiliations:** Department of Kinanthropology and Humanities, Charles University, Prague, Czechia

**Keywords:** Czechoslovakia, institute of physical education and sport, physical education and sport, state committee for physical education and sport, state office for physical education and sport

## Abstract

After February 1948, the new communist regime began to subordinate areas of life in Czechoslovakia to advocated political and ideological demands. Consequently, the unification of physical education and sports into Sokol was declared. In addition to institutional and legislative changes, the system of training for specialists was reorganized. The genesis of the Institute of Physical Education and Sport is reconstructed to illustrate organization and management of Czechoslovak physical education. The process of adopting the Soviet model of direct state control is described in the context of the geographic, historical, economic, and social specifics of a given territory. This research was based on the study of archival documents from the provenance of the State Committee and the State Office for Physical Education and Sport. This paper examines the intentions of the competent authorities, the circumstances of the foundation, mission, application of graduates and the content of studies at the Institute.

## 1 Introduction

At the funeral of Joseph Vissarionovich Stalin in Moscow on 9 March 1953, the Czechoslovak communist leader Klement Gottwald^1^ (1) was among the prominent attendees. His physicians had advised him against air travel, warning that his organism – severely weakened by alcoholism and untreated syphilis, as well as by an aortic aneurysm – was at significant risk. Gottwald disregarded this medical advice, and shortly after returning from Moscow, he died on 14 March 1953.

Whereas Stalin's death triggered a power struggle within the Politburo of Soviet Communist Party, no comparable political upheaval occurred in Czechoslovakia following Gottwald's death. He was succeeded in the presidential office by his close associate and incumbent Prime Minister Antonín Zápotocký^2^ (2). Meanwhile, Alexej Čepička^3^ (3), Gottwald's son-in-low, ceremonially inaugurated the Institute of Physical Education and Sport less than seven months later.

The aim of this study is to analyze the establishment of the Institute of Physical Education and Sport (hereafter IPES) in the broader context of the Sovietization of Czechoslovak physical education and sport after February 1948. Attention is paid to the political intentions behind the creation, its institutional framework, and its role in the centralized system of sport governance. The study also examines the significant transformation in the organization and management of Czechoslovak physical education and sport. This transformation was implemented by prominent members of the communist establishment active in the field of sport. From the perspective of Pierre Bourdieu's theory, we could define the process of shaping the form of university physical education by the relationship between the political and sports fields. For the period under examination, such a delineation is somewhat problematic. The period of the Sovietization of sport on which we focus was marked by power-driven terror aimed at the elimination of real, perceived, and eventually internal opponents (traitors) od communist policy. Late Stalinism deeply affected social relations in newly established communist dictatorships in Central Europe. This study examines this process using the example of Czechoslovakia. It introduced instability, uncertainty, and fear into society, including physical education and sport. These conditions were manifested in a high degree of arbitrariness. For this reason, our analysis focuses on the concrete reflection of broader societal processes in the organization and governance of Czechoslovak sport. We examined the relationship between communist officials’ conceptions and the new mission of university physical education and the specific needs it was intended to fulfill.

In Czechoslovak communist propaganda, Sovietization was embodied in the slogan “The Soviet Union – Our Model”, which became a kind of political mantra. Questioning it, even in the form of inquiries into its concrete meaning, was strongly discouraged. For the historian, the concept of Sovietization is therefore difficult to define precisely, as it was surrounded by an aura of unquestionable authority and near-sacred legitimacy. In this study, Sovietization is understood as a general characteristic of the broader social atmosphere. More specifically, it refers to the establishment of direct state control over physical education and sport. This control was centralized in the hands of the communist elite and exercised through state institutions.

Existing literature provides only a superficial description of the establishment of the Institute of Physical Education and Sport. Therefore, this research is based primarily on archival documents from the State Committee and the State Office for Physical Education and Sport related to its foundation. Our approach is predominantly inductive. We use archival evidence to reconstruct the motivations of individual actors and political mandates. We also identify the reasons behind the establishment of IPES as an independent institution subordinated to the chairman of the State Committee for Physical Education and Sport. The relatively short time span and the chronological sequence of legal measures resulted in the communist nationalization of physical education and sport, with the establishment of IPES justifiably regarded one of the specific outcomes of this process.

This article adopts predominantly chronological (diachronic) approach to analyze the establishment of IPES in broader context of Sovietization of Czechoslovak physical education and sport after February 1948. It first outlines the political and institutional transformation under communist rule, followed by an examination of the debates surrounding the creation of its foundation and early functioning. This approach allows us to reconstruct the sequence of institutional and legislative changes. It also reveals the political intentions and structural needs behind these developments. Furthermore, it helps explain how centralized state control was implemented and how it reflected broader mechanisms of communist power consolidation.

## 2 Changes in the organization of Czechoslovak physical education and sports implemented by the rulling communists

### 2.1 Czechoslovakia in the process of Sovietization of Central Europe after World War II

After World War II, Soviet leader Joseph Vissarionovich Stalin rigorously enforced the absolute submission of Central European countries to the policies of the Soviet Union. He relied on the presence of the Red Army, which had defeated the Nazi military forces in these regions. Between 1945 and 1949, in Hungary, Poland, Czechoslovakia, and the German Democratic Republic—formed from the Soviet occupation zone—communist parties, supported and gradually controlled by him, seized absolute political power.

The Soviet leadership created a chain of formally independent states that functioned as satellites of the Union of Soviet Socialist Republics (hereafter USSR). This system was intended to serve as a buffer zone against potential Western conflict. At the same time, the goal was to gain control over the industrial production capacities of the subjugated countries, thereby strengthening the geopolitical significance of the Soviet Union in Europe (4).^4^ After securing control over the political system and economy in Czechoslovakia, as in other Central European countries bound to Moscow, the next step was to subordinate additional areas of social life to the political and ideological demands of the ruling elites of the USSR.

The second stage of Sovietization in the so-called people’s democracies of Central Europe between 1948(1949) and 1953 is sometimes referred to as Stalinization, since both domestic and foreign policy in the Central and Southeastern European people’s democracies was decided by the all-powerful Generalissimo [for more (5)]. This period was marked by the consolidation of communist power within the political system, the army and police, state and regional bureaucracy, the press and radio, as well as education and culture. The main instrument of this process became unprecedented personnel transformations, in which real or alleged opponents of the communist regime, but also apolitical professionals, were replaced by so-called “people-cadres”. These individuals often lacked necessary professional qualifications, but immediately after the communist coup in February 1948^5^ had assured the communist their unreserved loyalty to the new regime.

As a part of the fight against the so-called class enemy, a series of political trials took place in the communist satellites, some of which – especially in Czechoslovakia – culminated in judicial murders. The process of Sovietization in Central Europe – including the scope of repression and its targeting of certain social groups – was neither uniform nor could it be. Social, economic, and demographic conditions varied too greatly between countries, and power struggles within the communist elites of the Soviet satellites unfolded with differing intensity and in different forms.

After February 1948, all industry in Czechoslovakia was nationalized, and peasants were obliged to enter agricultural cooperatives (6). Economic performance began to decline steadily, and a significant share of consumer goods, as well as a certain foodstuff, became available primarily through the black market. Any expression of discontent was rigorously suppressed under the guise of combating reactionary elements. The communist propaganda – disseminated through the press, radio, and newsreels – portrayed various aspects of social life in and overly idealized manner, intended to demonstrate the correctness of the path toward a prosperous communist future.

Sovetization naturally also extended to the sphere of physical education and sports. The organization and management of physical education and sports were transformed according to the Soviet system^6^. Their central effort was the rapid Sovietization of physical education and sports. Training of new physical education teachers, coaches, and professional officials followed one principle: strict adherence to the Soviet model.

The specific form of the path to the Sovietization of physical culture^7^ naturally varied depending on several factors. These primarily included the circumstances under which communists in each country achieved their fundamental political goal, i.e., to seize absolute political power. Additionally, the development of physical education and sports up to World War II, which resulted in a system of physical education and sports based on voluntary association activities in all the mentioned countries, influenced the convergence with Soviet system. This system was something the communists intended to fundamentally change. In detail, the outcomes of the Sovietization of physical education and sport were also influenced by the internal power struggles within communist parties. The Sovietization of sports in Central Europe should not be seen as the creation of an identical system for managing and controlling physical education and sports, i.e., establishing the same governing bodies and mechanisms for their operation. Every system, including totalitarian ones, must at least partially respect the geographic, historical, economic, and social context of a given territory. This context naturally varied significantly in the case of the Union of Soviet Socialist Republics (hereafter USSR) and the new so-called people's democracies of Central Europe. Moreover, in Czechoslovakia, none of the communist elite or physical education experts had any direct experience with Soviet physical education and sport. The Sovietization of society, including physical education and sports, in the communist Central European regimes during the late Stalinist period, became primarily a mantra that was not advisable to oppose. Some common features of the Sovietization of physical education and sport can be found across all so-called people's democratic regimes in Central Europe. Primarily, it involved subordinating physical education and sports to the political goals and visions of communist establishments and their strong ideologization.

In Central European states that fell under the control of the Soviet Union after World War II, the roots of voluntary association sports and physical education trace back to the second half of the 19th century. In East Germany, including the part managed by the Soviet army after the defeat of Nazism, as well as in Hungary, Poland, and Czechoslovakia, a system for the professional training of physical education organization instructors and university-level education for physical education teachers was established before World War II. Communists were thus able to use the material base and partly the content of the education after usurping political power. Physical education experts with university training and experience in sports organizations were able to teach at restored or newly established universities. Many of them had also taught physical education before the war. However, they had to demonstrate alignment with the goals of Communist Party policies and ideologies. Without meeting this condition, they could not hold positions at the new universities (7).

### 2.2 Unification and centralization of sports and physical education

Following the liberation of Czechoslovakia from Nazi occupation in May 1945, the country underwent a series of transformative changes across all facets of society. These changes largely aligned with the demands of the communists. President Edvard Beneš, who was expected to be a major supporter of the democratic faction, found himself under communist pressure. He struggled to maintain a balanced relationship in both domestic and foreign policies with the USSR and Western democracies. Beneš hoped to position Czechoslovakia as a mediator between the East and the West. However, Stalin dismissed such notions and pressed the Communist Party of Czechoslovakia (hereafter CPC) leadership to consolidate their power as quickly as possible.

The concept of creating an independent school for physical education and sports emerged in the specific socio-political milieu following World War II, shaped by the new direction of Czechoslovakia's domestic and international policies. The post-war evolution of physical education and sports in Czechoslovakia was greatly affected by the strategy adopted by the Communists to capture absolute political control.

The largest association that resumed its activities after the war was Sokol^8^. Sokol, founded in 1862, was not only the largest, but also the oldest gymnastics association. Its representatives emphasized the organization's national mission. Especially after establishment of Czechoslovakia in 1918, the Czech public perceived Sokol as a leading bearer of Czech patriotism. In 1947, it had 471,000 adult members [(8) p. 99]. Its leaders and members alike considered defending parliamentary democracy a fundamental responsibility. This stance made the Communists wary, as they perceived Sokol as a potential barrier to their goal of setting up a totalitarian regime. However, they could not directly confront the Sokol organization because it had a dense network of Sokol units, a large membership base, and enjoyed high moral standing among the Czech public. This reputation developed during the interwar period and strengthened during the Nazi occupation. Many Sokol members joined the resistance, and some were executed. The communists proposed merging all gymnastic clubs and sports unions into Sokol, aiming to diminish the effectiveness of the Czechoslovak Sokol Organization (hereafter CSO) leadership. They planned to gradually infiltrate the management of the overall organization and its subordinate structures, such as Sokol districts and units. Their efforts were unsuccessful until February 1948, but following the communist coup that month, they encountered no further obstacles to the planned consolidation of physical education and sports. The creation of a singular physical education and sports organization, Sokol, was declared. Following this consolidation, the Communists carried out extensive purges within Sokol at all levels. Leaders from Sokol were expelled, many lost their jobs, and some were imprisoned after rigged trials. The Communists placed their people in key positions within the CSO, as well as in Sokol districts and units, effectively taking control (9).^9^

The unification of all physical education and sports organizations into Sokol stemmed from the Communists’ immediate need for power consolidation. However, they were not content with this approach and aimed to adopt an organizational structure for physical education and sport from Soviet Union, where this sphere was at the time subject to full state control and central direction. After the February coup in 1948, the Communists focused on eliminating of their real and suspected adversaries, while also seeking to completely dominate the military and police. Consequently, significant changes in the governance of physical education and sports were only introduced at the turn of the 1940s to the 1950s.

In 1949, the Communist Party of Czechoslovakia initiated steps towards the direct state management of physical education and sports, emulating the framework established in other Central European countries under communist rule, inspired by the Soviet Union. In July of that year, the communist-dominated parliament passed the Act on state care for physical education and sport. This law heralded the formation of the State Committee for Physical Education and Sport, which was to comprise members from the “Czechoslovak Sokol Organization, national people's organizations, and distinguished experts in health, physical education, and sports”[(10) § 2, para. 1]. The procedural regulations for this state committee, which was envisioned as a collective body under state oversight rather than a purely state entity, were established in 1950. Despite this, the committee never commenced its functions. Instead, the operational body became the State Office for Physical Education and Sports (hereafter SOPES), designated as the “administrative office for issues related to physical education and sports” [(10) § 5, para. 1]. Entrusted with its leadership was Deputy Prime Minister General Ludvík Svoboda (11).^10^ The office primarily focused on administrative duties. However, in the absence of the state committee's activities, it was responsible, under the Secretariat of the Central Committee of the Communist Party of Czechoslovakia (hereafter CC CPC), for developing a system to manage physical education and sports. According to directives from the CPC leadership, this system was to ensure direct state control over physical education and sports through specific entities, bypassing involvement from other organizational representatives.

### 2.3 Mobilizing the masses: the need for socialist expertise

Representatives of the communist regime in Czechoslovakia, similar to their counterparts in the Soviet Union, highly valued physical education and sports for their importance in defense readiness, specifically in terms of preparation for military service.^11^ They also recognized their positive impact on public health, especially regarding the quality of the workforce, and the role of sports in representing the state internationally. Despite these high aspirations, there was a general shortage of educated professionals in state administration, training programs, and the military; this deficiency extended to the realm of sports as well. The aim was to transform sports based on the Soviet model and to encourage widespread participation among the working masses, which was expected to result in an increased number of record holders. However, there was a notable lack of coaches. During the interwar period, members of the middle classes predominantly led sports associations, and new training methodologies were largely introduced to Czechoslovakia by coaches from The Young Men's Christian Association. Meanwhile, Sokol instructors faced a “purification” process within their organization. Many were either expelled or chose to leave voluntarily, as they were unwilling to participate in activities that became heavily associated with the ideology and politics of the new political elites following February 1948. These experts were anyway considered undesirable by the Communist authorities. The Office of the Government, on October 4, 1950, outlined the positions that urgently needed to be filled by new physical education specialists: “There is an urgent need for experts for […] the unified organization Sokol, for people's administration (regional and district physical education instructors), for central authorities, apprenticeship homes, large factories, […] recreational centers of Revolutionary Trade Union Movement, as well as for the army, National Security Corps, and scientific research in physical education.” (12). The Office of the Government calculated the need for approximately two thousand physical education workers.

### 2.4 Soviet-inspired model of direct state control

In the autumn of 1951, the leadership of the CPC deemed it is the right time for a significant organizational shift in the management of physical education. Inspired by the Soviet Union's model, this shift aimed to bring about the complete Sovietization of sports through direct state control. In this system, state agencies would eagerly comply with all instructions from the Central Committee of the CPC and its secretariat. This is evidenced by an undated and marked as highly confidential document from the office of a member of the CC CPC- Jiří Hendrych.^12^ Containing only slight alterations, this document reproduces a proposal that was sent to Hendrych by the presidency of the political and organizational secretariat of the Central Committee of the CPC on January 15, 1952. The proposal's working title was *Proposal for Organizing Physical Education and Sport in the Czechoslovak Socialist Republic*. *Based on the Experiences from the Soviet Union.* In the document, Hendrych clarifies that “the focus is not on reorganizing the Institute for Physical Education and Sports but on how we intend to implement experiences from the Soviet Union in general.” (13). He further notes that “the key initiative is the establishment of a state committee for physical education and sports.” (13).

Whenever a decision was made by the Secretariat of the Central Committee of the Communist Party, it was the responsibility of the parliament or the government to formalize this decision into law. Additionally, the government had to ensure that these political decisions from the Communist Party were implemented effectively through legal regulations. This process was also applied to a fundamental organizational change in the governance of physical education and sports, which effectively eliminated the last remnants of association-based physical education by dissolving the unified physical education organization Sokol. The Act on the Organization of Physical Education and Sport, which came into effect on December 17, 1952, not only regulated but also redefined the management of physical education and sports. The Sovietization of physical education and sports, or rather the fulfillment of the Czechoslovak communist leaders’ vision for their Sovietization, was completed. Starting from Christmas 1952, the State Committee for Physical Education and Sport (hereafter SCPES) under the government of the Czechoslovak Republic has been implementing ’state direction and control of physical education and sport. The chairman of SCPES was “appointed and dismissed by the president of the republic upon the proposal of the government” [(14) § 3, para. 1].

The State Committee became the exclusive and ultimate authority in physical education and sports management. It not only directed this sector of society but also monitored that physical education and sports aligned with the policies and ideology of the CPC, as dictated by the Central Committee comrade members. The State Committee operated through branches within every level of government—namely, the Regional and District National Committees (15). These branches were responsible for implementing the State Committee's directives across Czechoslovakia, always in accordance with the Communist Party's policies.

The first chairman of the SCPES was appointed by the President of the Republic, which enhanced his position and authority. This position was held by František Janda^13^. It is not clear from the existing documents whether appointing him as the chairman of SCPES marked the beginning of his fall from influential military positions or, on the contrary, if the leadership of the Communist Party considered physical education and sports to be of such significant defense importance that they selected a man with a wealth of experience from the state's repressive forces, expecting him to use his organizational abilities and show decisiveness in carrying out party orders. Nevertheless, František Janda was a very active chairman of SCPES, and his role in Sovietizing Czechoslovak physical education and sports was certainly significant. By the end of 1953, following a series of internal power struggles within the Communist Party of Czechoslovakia, which impacted the governmental structures, he became a persona non grata. Evidence of this is a letter from the government's presidency to him dated November 19, 1953, which states that ‘the principles of state discipline are being breached in the operations of the State Committee for Physical Education and Sport (16).

Through the enactment of the Physical Education and Sports Law in 1952, the leadership of the Communist Party of Czechoslovakia decided to link physical education to the workplace. This decision was grounded in the theory of Marxism-Leninism, which posited that the working class should be the main support base for the CPC's power position. Consequently, the law on the organization of physical education and sports stipulated that “the implementation of physical education and sports is principally organized according to the workplace” and that “voluntary physical education and sports are conducted in physical education and sports organizations, particularly created within the Revolutionary Trade Union Movement” [(14) § 9, para. 1]. Sports clubs in manufacturing plants, offices, and vocational schools were expected to continue their activities under the supervision of the Revolutionary Trade Union Movement. This arrangement placed sports under the direct oversight of the CPC, as nearly every workplace hosted a basic CPC unit. These members monitored and reported bourgeois or anti-socialist attitudes. Their surveillance extended beyond sports into broader social life.

## 3 Exploring new forms of higher education in physical education and sports in Czechoslovakia after World War II

### 3.1 Roots of Czechoslovak physical education teachers training

The document from SCPES addressed to the Office of the Government Presidency titled *Proposal for a Government Resolution on the Establishment of the Institute for Physical Education in Prague*, which justified the need for the expedited establishment of the Institute, states that “besides Albania, Czechoslovakia is the last people's democratic state that has not yet built an educational institution for the training of [..] specialists in extracurricular physical education” (17). It continues with an argument that no one dared to trivialize or ignore: “Especially in the USSR, great care was devoted to the proper study of physical education” (17). This indicates a comparatively delayed process of institutionalization relative to other Eastern Bloc countries. This disparity was particularly evident in comparison with the German Democratic Republic [see (18)].^14^

Although a separate college for physical education and sports did not emerge in Czechoslovakia until the 1950s, the university-level training of secondary school physical education teachers had a long-standing tradition in the Czech lands. In 1892, the Prague Charles-Ferdinand University was divided into Czech and German institutions (19). That same year, a two-year *Educational Course for Physical Education Teaching Candidates at Secondary Schools and Teacher Training Institutes* commenced in Prague. A candidate for teaching physical education at secondary schools had to complete a second subject at the Faculty of Philosophy, which included the study of natural sciences until 1920. Since the study of philosophical and natural science subjects took at least three years (6 semesters), candidates for physical education teaching were subject to an asymmetric model of higher education. During the interwar period in Czechoslovakia, the imbalance gradually vanished as the length of the educational program was progressively increased to six semesters in 1920 and further to eight semesters in 1936 (20). In 1935, the parliament enacted a law for the creation of an independent physical education institution - the Tyrš State Institute of Physical Education (21). Nonetheless, the realization of this law was obstructed by the absence of financial means in the state budget after World War II ended.

After the end of World War II, the activities of universities were resumed on 22 October 1945.^15^ At the same time, a reform of higher education for teachers was being prepared. In November 1945, the President of the Republic, Edvard Beneš, issued a decree *On the Education of Teachers*, whose first paragraph stipulated that “teachers of all types and levels shall be educated at pedagogical and other faculties of higher education institutions” [(22) para. 1]. The preparation of physical education teachers for teaching at both primary and secondary schools fell under the responsibility of the pedagogical faculties. The Communists agreed with this, and consequently, by law No. 100/1946 Coll., “pedagogical faculties were established at all universities (Prague, Brno, Bratislava)” whose task was “to cultivate pedagogical sciences and educate candidates for teaching positions at schools of all levels” (23). By government decree No. 170/1946, the duration of study for elementary and middle school to six semesters. The candidates for teaching at high schools faced a study period of 8–10 semesters (24). Pedagogical Faculty in Prague was the first to open its doors in November 1946.

### 3.2 Reshaping the debate over independent institution

Following the communists’ rise to absolute power in February 1948, almost everything that had worked before was distrusted by the new ruling elite. This led to changes in all aspects of society, therefore in physical education and sports as well, regardless of whether these changes were necessary or logically grounded. Where structures remained, they were infused with entirely new meanings. Education, including higher education, received special focus. The communists believed socialism required a new type of person. This individual had to reject capitalist values and fully embrace socialist ideology. This approach would guide them like a compass toward a happy communist future. Schools played a crucial role in this transformation because their influence could be directly overseen by party and state authorities, unlike family upbringing. Nonetheless, the education of future high school teachers, including those teaching physical education, saw no significant organizational changes. Pedagogical faculties continued their operations with their university nature intact.

In May 1950, a new Act on Higher Education was adopted. Its second paragraph stipulated that universities were to “educate highly qualified specialists both professionally and politically, loyal to the people’s democratic republic and devoted to the idea of socialism” (25). Political loyalty thus became a fundamental attribute of a university student. Members of the highest leadership of the CPC generally approached university educated individuals with distrust, a perception likely reinforced by the fact that the vast majority of party leaders themselves had never attained a university degree.^16^ They nonetheless understood the need for educated professionals, particularly in technical and medical fields, but remained convinced that especially those who had studied at universities in interwar Czechoslovakia might be inclined toward what was labeled bourgeois values. Graduates of universities in so-called socialist Czechoslovakia, by contrast, were expected – or rather required – to be different. They were supposed to match, and ideally surpass, the expertise of their old counterparts, but above all to embody the ideal of the new socialist person. This notion, like other negative labels (e.g., reactionary) or positive ones (e.g., builders of communism), was deliberately defined in vague terms so that it could be flexibly interpreted according to immediate political and ideological needs. In the context of Sovietization of a relatively advanced economic and cultural Czechoslovak society, the concept of new socialist person represented a social construct, a symbol of transformation, and an individual role model (26).

The aforementioned Higher Education Act of 1950 placed universities under direct state supervision: “The supreme administration of universities and oversight of them belong to the Minister of Education, Science and Arts.” Moreover, the government was empowered to establish, abolish, or merge universities and individual faculties by decree (25). Higher education was thus centrally subordinated to the relevant ministry, which could intervene directly in university affairs. The content of education was further controlled by CPC organizations established at both the university and faculty levels. After the communist seizure of power in February 1948, an extensive purge was carried out at universities, particularly at faculties focused on social sciences, resulting in the expulsion of numerous teachers and students. Lecturers who failed to publicly declare their support for CPC policy after February were deemed politically unreliable and therefore could no longer hold academic positions. Student who had been active in non-communist youth organizations, or who had openly voiced disagreement with the practices of the new regime, were expelled as well.

Many were also excluded because of their so-called bourgeois origin. This social category was never precisely defined, leaving considerable room for arbitrariness and the settling of personal scores. For the communist elites, expertise was understood in strictly utilitarian terms – that is, as the acquisition of knowledge and skills necessary for the performance of a given profession. Creativity arising from independent thought, by contrast, was perceived as an expression of individualism, wholly incompatible with collectivism imbued with the determination to follow the directives and demands of the Communist Party.

From that point onward, applicants from so-called bourgeois and petty-bourgeois families were no longer to be admitted to universities. In 1949, reviews were carried out at Charles University in Prague and Masaryk University in Brno, after which many students – amounting in some faculties to several tens of percent – were expelled. At Charles University, law students were the most severely affected, with 45 percent of the total body being excluded (20). Between 1948 and 1950, the teaching of Marxism-Leninism was introduced at all university faculties (20). By restricting access to higher education for intelligent and talented young people from middle-class families, the educational potential of society was partially undermined. In admission to university study, the so-called class criterion was applied most rigorously in the social sciences, including teacher training. In the technical fields, by contrast, family social background played a lesser role. Some expelled students, as well as applicants for so-called bourgeois families, were able to redeem themselves through work in manual labor professions and were then admitted to university studies.

The training of specialists designated to work in the area of extracurricular physical education and sports was to be entrusted to the teaching staff at a newly created independent university of physical education. The staff of the CPC secretariat, representatives of the Ministry of Education, Science and Arts, and SCPES debated the form of the new school. One option was to establish Faculty of Physical Education at Charles University. However, the leadership of SCPES clearly preferred the establishment of an independent college, as it would have the “advantage of flexibility in educational and scientific […] work” and further direct cooperation “with unified physical education”, and the management and teachers of the school would have the opportunity “to directly establish international cooperation” (27). The arguments were well-founded, yet it's also plausible that the SCPES's top officials were keen on taking full control over both establishing and managing the school. The concept of a unique state body directly overseeing the university likely appealed to members of the CPC secretariat. This approach would align with the era's overarching principle of adhering to the Soviet model.

The implementation of proposals of leading representatives of SCPES was complicated by the distrust that proponents of hardline Sovietization of political and social life in Czechoslovakia harbored towards the committee's chairman, Ludvík Svoboda. They criticized him for not conducting a thorough purge within the officer corps while he was the Minister of Defense. In April 1950, he was removed from his position as Minister of National Defense and appointed as Deputy Prime Minister, tasked only with leading the State Committee for Physical Education and Sport, which represented a political demotion. By September 1951, he was relieved from this role as well [(11) p. 110–145]. During communist regime, dismissals were typically justified by vague allegations of inadequate performance, while specific reasons were seldom provided. In this context, Ludvík Svoboda's dismissal was officially attributed to the poor performance of the SCPES. At the same time, the establishment of a higher education institution for physical education was intended to constitute another component of the newly emerging organizational structure.

### 3.3 The emergence of the Institute of Physical Education and Sport

In the spring of 1951, a commission was established with representatives from the Ministry of Education, Science, and Arts, the SCPES, and the Prague Pedagogical Faculty. This commission assessed the professional level of students and teachers at the Institutes of Physical Education and Sport at the Pedagogical Faculties in Prague, Brno, Bratislava, and Olomouc, as well as their political awareness. The commission members were dissatisfied with the political profiles of students in the upper years, “especially regarding class background and political awareness” (28). They attributed it to the fact that “apolitically oriented athletes and members of the Sokol movement went to study physical education,” which corresponded to the reality that these were students who began their studies before the communist coup in February 1948. The commission noted that they were “often specialists in a narrow area of physical activities but had a limited appreciation for discipline and collective work” (28). Graduates from workers’ preparatory courses, who received proper political training and understood what was expected of them, were found to be the best equipped politically. The commission reported that the first-year students had a satisfactory class background, although their selection during the admissions process “could not be as strict as physical education would require” because “the admissions committees were trying to meet target numbers” (28). In the 1950s, it was not easy to find candidates for university study, especially in physical education, who ideally came from a working-class family, wanted to build communism, and simultaneously possessed the intellectual and, in the case of physical education, also the physical capabilities necessary for university studies.

The members of the commission were not satisfied with the teachers either, noting that “conscious members of the Communist Party of Czechoslovakia constitute a minority at the institutes.” Those who were non-partisan, meaning not members of the CPC, according to the commission, were not particularly enthusiastic about communism. The commission concluded that it would be very difficult to eradicate remnants of the past in them and questioned “whether their current positive attitude is genuinely sincere” (28). Despite identifying political shortcomings among some students and teachers in physical education departments of pedagogical faculties, the commission members recommended “to retain the study of physical education for secondary and higher secondary school teachers at pedagogical faculties” (28). The primary reason for their recommendation was the “significant shortage of physical education teachers.” Furthermore, they advised the establishment of a higher education institution in 1952 to facilitate the “training of highly qualified personnel in extracurricular physical education and to promote the development of scientific expertise in the field of physical education” (28).

In 1952, the party elites were content with the introduction of a new system for state governance and the structuring of physical education and sports, modeled after the Soviet example. The most significant issue at the time was the scarcity of coaches, administrative staff, and sports instructors, particularly for the military and the National Security Corps.^17^ Therefore, the law on the organization of physical education and sport also entrusted the State Committee with the “supreme administration and supervision over higher physical education schools” (14). At the end of the year, there was yet nothing to supervise, but that was set to change quickly because the government could establish the “university of physical education” by its decree. On April 29th 1953, a very succinct government decree was issued, establishing the Institute of Physical Education and Sport in Prague. The decree declared that the institute was a higher education institution and was led by a rector and the institute's council. The chairman of the SCPES was responsible for implementing the decree (29).

### 3.4 Institute management and student's perspective

The Institute of Physical Education and Sport was ceremoniously opened on October 1, 1953. Dignitaries in attendance included the Minister of National Defense Alexej Čepička. General Colonel František Janda, the Chairman of SCPES; and Ladislav Štol, the Minister of Education and Culture, attended as well. They were joined by Ladislav Serbus, who had been recently appointed as the rector of the Institute. Since July 1950, Serbus had worked on a commission directed by the SCPES to develop the curriculum for this new higher education institution specializing in physical education (30). President Antonín Zápotocký officially appointed him as rector on May 11, 1953.

In their quest to find suitable candidates for the academic body, the Communists established two fundamental criteria: political reliability, meaning alignment with the political and ideological objectives of physical education and sports as delineated by the CC CPC, and professional competence in physical education and sports, demonstrated through education and experience. They succeeded in finding, at least for the management of IPES, communists who had acquired physical education training and practice during the interwar period, joined the CPC before February 1948, and had a record of resistance activity against the occupiers in their resumes, though often not very specifically detailed. Ladislav Serbus was one of them. From a young age, he was actively involved in the Sokol movement, where he first trained as a regular member in the pupils’, youth, and adult categories, and later served as an instructor. Between the years 1926 and 1931, he completed an Educational course for physical education candidates at secondary schools and teacher training institutes, alongside another major in chemistry, and worked as a high school physical education teacher. After World War II, he taught physical education at the Faculty of Education in Prague, and in 1951, he was appointed Associate Professor in the field of theory and methodology of gymnastics. He joined the Communist Party shortly after the liberation of Czechoslovakia) [(31) p. 5]. He was loyal both politically and professionally—we can say that he was a physical education instructor in every regard. During the occupation of the Czech lands, after the establishment of the Curatorium for the Re-education of Czech Youth in 1940, he led the Department of Basic Training [(32) p. 63]. There is no evidence in existing literature or known documents to suggest that Ladislav Serbus was involved in ideological education aligned with National Socialism, nor was he penalized for his activities related to the Curatorium after the war. Nevertheless, it is plausible that members of the Central Committee of the Communist Party were informed of his involvement and kept this information as a possible leverage against the IPES rector in the event of any future “disobedience”.

The second man in the leadership of IPES was Jan Libenský who had previously served as the assistant director of the Institute of Physical Education of the Pedagogical Faculty at Charles University from 1951 to 1953. Born into a working-class family, Libenský completed his high school education in 1922 and pursued studies in natural sciences, geography, and physical education at the Faculty of Science of Masaryk University in Brno. From the early 1930s until 1939, he worked as a teacher at a secondary school in Žilina, and during the war, he continued teaching in Prague. His career was advanced by his appointment as the regional school inspector by the Minister of Education and Culture, Zdeněk Nejedlý, in May 1946. When this position was abolished in 1949, he became the head of the department for grammar schools at the Ministry of Education. He joined the Communist Party of Czechoslovakia after February 1948, but according to his biography, he had sympathized with its policies even before the Second World War. In July 1953, Jan Libenský was appointed as an associate professor for the field of theory and methodology of physical education. That same year, he joined the Institute of Physical Education and Sport, where he took charge of the Department of Theory of Physical Education and served as Vice-Rector for pedagogical and political education work [(33) p. 11–13].

Within the faculty, several notable personalities were present. For instance, the Department of Athletics was led by Karel Kněnický, a versatile athlete who represented Czechoslovakia at the Olympic Games in Amsterdam in 1928 and in Berlin in 1936. Since the Institute's foundation, young assistants who had to complete their physical education studies after World War II, due to the closure of Czech universities by the Nazis in 1939, also worked there. According to a memoir published by Jan Libenský, I. G. Čudinov played a crucial role in establishing IPES. In preparation for the Institute's curriculum, a request was made to the SCPES for the assignment of an academic official from one of the Soviet physical education institutions. I. G. Čudinov, deputy director of the Central Institute of Physical Education in Moscow, arrived in Prague in the spring of 1953 and made a significant contribution to the educational concept.

The Act on the Organization of Physical Education and Sports, passed in December 1952, led to the dissolution of the Sokol organization. The Act introduced an entirely new organizational structure and system of governance for physical education and sport. As a result of this legislation, Sokol as an organization and legal entity ceased to exist. As a result, their headquarters, the Tyrš House—a complex in the center of Prague featuring a sports field, gym, swimming pool, and student accommodations—was made available. In this context, Jan Libenský recognized the efforts of SCPES Chairman František Janda. Janda played a key role in acquiring this facility for the IPES and secured 5 million Czech crowns for the “essential adaptation” of the sports facilities and classrooms [(34) p. 17]. It is important to note, however, that the task of securing these material resources was assigned to him as SCPES chairman by a government decree regarding the establishment of IPES. The Institute began its operations in the academic year of 1953/54 with an inaugural year-long coaching school.

In April 1953, Regional Committees for Physical Education and Sport received a letter titled *Recruitment of Students for the First Year of the Institute of Physical Education and Sport* from the Vice President of the SCPES - Vilém Mucha.^18^ The document, among other things, outlined the job positions for which the students of the Institute were being prepared. These positions included: “physical education teachers at universities, scientific-research workers, […] leading officials in the physical education movement, professional sports coaches, and teachers at sports schools” (35). On May 22, 1953, Alexej Čepička, the Deputy Prime Minister and Minister of National Defense, officially signed the founding charter of the Institute of Physical Education and Sport (36).

Graduates of IPES were also expected to serve as guarantors of professional competence for regional and district committees on physical education and sports. Graduates were guaranteed employment because the system needed educated and politically reliable professionals. However, this did not imply that an Institute graduate, like graduates from other universities, had the freedom to choose their place of employment. In the 1950s, university graduates were assigned a so-called job appointment, which directed the new diploma recipients to a specific factory, office, hospital, etc., where they were obligated to remain for a period of 3–5 years.^19^

Candidates applying to the IPES faced stringent criteria, encompassing not only their “political awareness” but also their academic achievements as evidenced by their high school records. Additionally, they were required to demonstrate a high level of proficiency in sports during the practical entrance examination. Applications for the IPES were submitted through high schools, the Regional Committee for Physical Education and Sport, and the SCPES. An endorsement from either the school, regional committee, or SCPES had to be supported by comprehensive “cadre material”.^20^ While not mandatory for admission, membership in the CPC was likely beneficial during the selection process.

The curriculum of the institute included history and theory of physical education, pedagogy, psychology, biomedical subjects such as anatomy, physiology, biomechanics, hygiene, and first aid. Students of IPES also had to study Marxism-Leninism, including Soviet experiences, and Russian language. Assessing the extent to which teaching and daily life at IPES were influenced by Stalinist policies and the ideology of the Communist Party of Czechoslovakia in the 1950s is challenging. Undoubtedly, there were significant external manifestations. The initial years at IPES were marked by a semi-military regime. Students were required to live in dormitories, wear uniform tracksuits, and attend daily roll calls. In terms of curriculum content, Marxism-Leninism was predominant or ranked second only to pedagogical practice (in 1953, Marxism-Leninism accounted for 444 h, and pedagogical practice for 540 h). Among practical subjects, gymnastics was attended the most (322 h), possibly because the head of the gymnastics department was the rector of the institute, Ladislav Serbus (34). The teaching of subjects within the social sciences domain, particularly theory and history of physical education and sports, as well as pedagogy, was mandated to align with the works of Soviet scholars. This directive influenced both the choice and interpretation of factual knowledge. However, the content of biomedical and practical subjects was likely less tainted by ideological influences. For physical education teachers, akin to doctors, maintaining a professional identity was paramount. Many teachers likely viewed ideological declarations as a necessary compromise. This allowed them to preserve educational quality and partial institutional autonomy. For mid-generation academics who had been teaching physical education in high schools before and during World War II, joining IPES marked a significant leap in their careers, sometimes culminating in professorship. For younger assistants, who pursued physical education studies post-war, securing a role at IPES presented an opportunity for a promising university career.

## 4 Conclusion

The foundation of the Institute of Physical Education and Sport marked the culmination of efforts by two generations of gymnasts and athletes who, since 1919, had been advocating for the establishment of a dedicated higher education institution for physical education. Influenced by the advocacy emphasizing the health benefits of physical exercise, the parliament decided to establish a higher institution for physical education. However, the enactment of the relevant law occurred at a time when the national budget was grappling with the aftermath of the great economic crisis, leading to the non-fulfillment of the law's provisions. It is somewhat paradoxical that the dream of interwar physical educators, who were deeply democratic at heart and mostly leaders within the Sokol movement, came to fruition during an era dominated by the peak of Stalinism. Following the communist takeover in February 1948, however, the establishment of an independent physical education institution emerged as one of the political priorities for the new rulers. This urgency was driven by a pressing need for qualified specialists whose higher education would encompass not just their professional expertise but also, and more importantly, ideological indoctrination. This training was designed to dogmatically instill views and attitudes in young people, aligning them with the policies of both domestic and Soviet communists. Furthermore, the existence of several higher education institutions for physical education in the Soviet Union provided a strong justification for founding IPES, as emulating the Soviet model became a fundamental imperative in European countries where communists had seized power in the 1950s.

The Institute of Physical Education and Sport, whose graduates were expected to serve as leading organizers – what in today's terms might be called managers – began to train and supply specialists for work at all levels of sporting life. They were prepared to become lecturers of physical education and sport at university departments, and as officer-instructors of physical training in the army. The regime presented them as one of the showcases of the Communist Party's care for physical education and sport. At the rhetorical level, its leadership consistently a demonstratively adhered to Soviet model. The curriculum continued to include generously allotted hours of Marxism-Leninism.

For many students and teachers alike, however, sport itself – understood as an activity involving physical abilities and motor skills directed toward performance – constituted the primary, and in many cases the only, meaningful identity at IPES. Popular practical training, along with logically related subjects concerning human body and its functions (such as anatomy and physiology), formed for them the substantive core of the educational program. The remainder of the curriculum was perceived by students, who had applied to the Institute because of their passion for sport – most of them being active athletes themselves – merely as complement to their education. Courses in Marxism-Leninism were, for most, regarded more as an unavoidable burden.

At IPES, as in sport more broadly, the capacity for perseverance and the will to overcome obstacles were highly valued. Pride in being student of the Institute, and the accompanying sense of belonging, did not arise from the expectation of mastering every discipline. It was rather dependent on achieving a certain level of competence and demonstrating the ability to perform even in sports that were not one's strengths.

With the establishment of the Institute of Physical Education and Sport, a distinct and long-lasting educational tradition was formed. In practical subjects, instruction closely resembled sports training itself, as it was structured around the fulfillment of performance standards and measurable outputs. In biomedical disciplines, as well as in subjects such as the history of physical education and sport, the emphasis was placed on the acquisition of an extensive body of prescribed knowledge.

This model of education, shaped under conditions of centralized state control and ideological supervision, allowed only limited space for students’ independent intellectual initiative, critical reflection, or methodological innovation. Nevertheless, it became structurally embedded in the training of physical education specialists and influenced the character of university-level sport education for decades.

Future research should therefore examine how this educational paradigm evolved in response to broader societal transformation during 1960s-1980s and, in particular, how it was reassessed, or dismantled after the collapse of the communist regime in 1989.

## Footnotes

^1^Klement Gottwald (1896-1953), Czechoslovak communist politician. From 1929, he served as a member of National Assembly. He was Prime Minister of Czechoslovakia from 1946 to 1948 and, following the communist coup, was elected President of Czechoslovakia.

^2^Antonín Zápotocký (1910-1957), Czechoslovak communist politician and trade union leader. A founding member of the Communist Party of Czechoslovakia, he was active in the labor movement and imprisoned during World War II in the Sachsenhausen concentration camp. After the war, he served as Prime Minister of Czechoslovakia from 1948 to 1953 and subsequently as President of Czechoslovakia from 1953 until his death in 1957.

^3^Alexej Čepička (1910-1990), a Czechoslovak politician. From 1942 to 1945 imprisoned in Auschwitz and Buchenwald concentration camps. In 1947, he married the daughter of the communist leader Klement Gottwald and served in high government positions (Ministry of Trade, Ministry of Justice, Ministry of Defence) and party positions until 1956. He played a significant role in the dissolution of churches and in purges within unified Sokol organization.

^4^The subordination of the economies of the so-called people's democracies to the economic interests of the USSR was institutionalized through the Council for Mutual Assistance (Comecon), established in January 1949. Its founding members, alongside the Soviet Union, were Bulgaria, Czechoslovakia, Hungary, Poland and Romania. See: Kramer M. *Stalin, Soviet Policy, and the Establishment of a Communist Bloc 1941-1949*.

^5^In February 1948, the Communist Party exploited the resignation of several non-Communist ministers from the Czechoslovak government. Under intense political pressure from Klement Gottwald and amid mass mobilization orchestrated by the Communists, President Edvard Beneš was compelled to accept their resignations, refrain from calling early elections, and appoint new ministers in accordance witch Communist proposals. At the same time, the secret police began arresting representatives of democratic political parties.

^6^The Soviet system of physical education and sport, based on a model of direct state control, in which all structures strictly and obediently executed directives issued by the leadership of the Communist Party.

^7^In this paper, physical culture is understood in a broad sense as an umbrella term for socially and historically shaped bodily practices and discourses, while physical education is understood as a formally organized educational practice, and sport, understood as institutionalized competitive physical activity governed by rules and oriented towards performance.

^8^Sokol was founded in Prague (Czech lands of the Habsburg Monarchy) in 1862 as a voluntary gymnastics movement, influenced in part by contemporary German Turnern. Its core purpose was structured physical training and civic-moral education through a distinctive system of gymnastics and mass exercises, and it became a key pillar of modern physical education and sport in the Czech lands and later Czechoslovakia. Sokol should be understood priamrily as a gymnastic systém and organization rather than a political association in the narrow sense.

^9^Waic, M. *Sport in Czechoslovakia 1945-1989*.

^10^Ludvík Svoboda (1895-1979), Czechoslovak soldier and politician. During World War II, Svoboda led the 1st Czechoslovak Army Corps on the Eastern Front in collaboration with the Red Army. After the liberation of Czechoslovakia, he served as the Minister of National Defence from 1945 to 1950.

^11^In March 1949, a defence law was passed that established conscription for all men from 17 to 60 years of age and set the duration of mandatory basic military service at 12 months. Later, the period was extended to 24 months.

^12^Jiří Hendrych (1913-1979), a Czechoslovak post-war politician, a member of Central Committee of the Communist Party of Czechoslovakia, a member of the National Assembly of the Czechoslovak Republic. One of the most influential members of the broader CPC leadership.

^13^František Janda (1898-1964), Czechoslovak general. Janda was promoted to the rank of general in April 1948, likely because he oversaw the arrest of politicians from non-communist parties during the communist coup in February. From December 1946 to October 1948, he served as the head of the III. Department at the Ministry of the Interior, meaning he was the chief commander of the National Security Corps. Afterwards, he returned to the army, where his military cereer culminated in the position of commander to the first military district in Prague.

^14^On the situation in the GDR.

^15^The closure of Czech universities on 17 November 1939 occured based on a decree issued by the Reich Protector Konstantin von Neurath. The pretext for the Nazi reprisals was several autumn student demonstrations against the Nazi occupation of Czechoslovakia, class of 1935.

^16^Of the thirteen men elected to the Presidium of the Central Committee of the Communist Party of Czechoslovakia by the Party's 8th Congress in March 1946 (with only one woman among them), only one had completed a law degree at Charles University, while another left his studies unfinished. Most of them came from modest social background where pursuing higher education was not an option. It should be noted, however, that they possessed extensive life and political experiences. They belonged to a generation born at the end of the nineteenth or the beginning of the twentieth century. Some of them had fought in the First World War, all had been engaged in political activity within the CPC or related organizations during the interwar period. Majority had taken part in the resistance against Nazism during the Second World War.

^17^NSC consisted of various police units and the Border Guard.

^18^Vilém Mucha (1892-1973), Czechoslovak sports official and a member of the Communist Party of Czechoslovakia. One of the founders of the Federation of Worker's Sports Associations. From 1950 to 1952, Mucha served as the Deputy Chairman of the State Office for Physical Education and Sport. From 1951 to 1954 he was the Chairman of the Czechoslovak Olympic Committee.

^19^These job assignments frequently required school graduates to relocate away from their homes, often to border regions that had become depopulated following the explusion of German residents in 1945-1946. The factory, office, or other establishments to which the graduates were assigned were obligaded to provide housing.

^20^The document did not have a specific format but included information such as, origins’ (worker, craftsman, peasant) based on the original profession of the parents. It also highlighted the applicant's active participation, for instance, membership in youth organizations, the Czechoslovak-Soviet Friendship Society, and any positions held within these groups. A general assesment was also provided, e.g., posseses a favourable view towards the people's democratic system.‘ If it was indicated that the individual did not share this view, they were not accepted.
